# The clinical utility and outcomes of microwave ablation for colorectal cancer liver metastases

**DOI:** 10.18632/oncotarget.15244

**Published:** 2017-02-09

**Authors:** Pengyuan Song, Lijun Sheng, Yahong Sun, Yuji An, Ya Guo, Yafei Zhang

**Affiliations:** ^1^ Department of Oncology, Affiliated Hospital of Shandong Academy of Medical Sciences, Jinan, Shandong, P.R. China

**Keywords:** MWA, CRLM, DFS, OS

## Abstract

In recent years, the microwave ablation (MWA) has been reported to play an important role in the treatment of patients with colorectal liver metastases (CRLM). In this work, 62 cases of patients who received MWA for liver metastases from colon or rectal cancer between Jan 2012 and Jan 2014 were enrolled in this trial. 28 underwent MWA, and 34 were treated with liver resection as control. Perioperative and 60 months of follow-up data were collected to analyze potential adverse effects, concurrent conditions and survival status. Here, we found there were no significant differences between both groups in the baseline level, including gender, size, number and pathological type (all *p*>0.05). In those patients, the mean hospitalization duration of patients with MWA is 5.9±0.9d, which is significantly different from control (11.8±6.9 d) (*p*<0.001). Little severe complication was observed in MWA group, while 26.5% (9/34) of patients developed severe complications (*p*=0.003). Besides, the mean hospitalization cost of patients with MWA was significantly less than that of control (*p*<0.000). Additionally, we found no statistically significant differences in disease-free survival (DFS) (*p*=0.156) or overall survival (OS) (*p*=0.580). In conclusion, MWA may be a safe, economical and competent way to treat inoperable CRLM patients, which has more advantages than liver resection in some degree.

## INTRODUCTION

Colorectal cancer (CRC) acts as the most common one in all malignant tumors in the worldwide. In the clinical practice, advanced CRC patients can develop into CRLM stage [1, 2]. Operative liver resection of CRLM was selected to treat and promote the overall survival of CRLM patients, while about 21% of CRLM patients can endure surgical resection owing to potential adverse effects [3]. Based on clinical practice guidelines of National Comprehensive Cancer Network (NCCN), liver resection is suggested to be the first option since curative resection is benefit to CRLM patients, however, no putative and strict standards are used to define the operation standard of CRLM patients. Traditionally, CRLM with the size more than 5cm cannot be treated using liver resection, thus CRLM with the size more than 5cm is defined as unresectable CRLM [4, 5]. Therefore, it is critical to identify some useful and novel ways to treat unresectable CRLM.

As reported, long-term therapy outcome after patients undergoing radiofrequency ablation (RFA) is satisfactory [4–6]. RFA has been reported as a safe and effective tool to treat cancer patients, while RFA also has its limitations, including an increase of impedance at 100 °C, a limited and small region of active heating, a decrease of effectiveness with charring, and limited multiple antennae [7]. It should be noted that MWA, a kind of thermal ablation therapy, does not rely on electricity current, and keeps effective when its temperatures are more than 100°C. On the other hand, MWA is equipped with many kinds of antennae, which can generate more larger ablation zone than RFA [8, 9]. Microwaves are featured as high temperatures, very short time and large ablation zones, which can improve treatment efficiency and broaden its ablation zones, and has no obvious heat-sink effects [10]. In recent decades, MWA is emerging as a kind of valuable and alternative way to treat hepatic diseases [11].

In the present study, we investigated outcomes (local and systemic recurrence) and survival status of CRLM patients who were treated with MWA at our department. We assumed that MWA can be a safe and effective way to treat colorectal cancer liver metastases. Furthermore, we try to determine the average hospitalization periods and hospitalization costs.

## RESULTS

### Patient demographics and baseline characteristics

**Figure 1:**
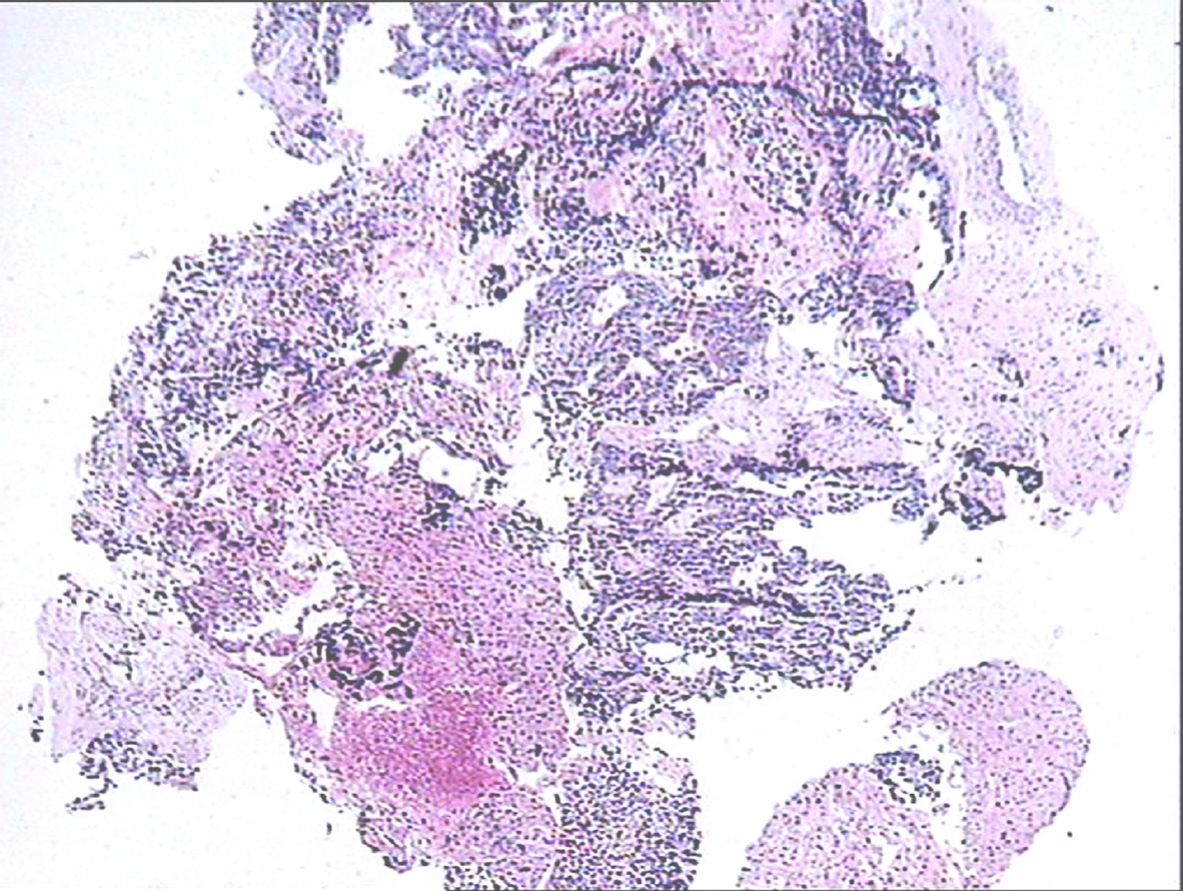
Pathological identification of CRLM HE staining was conducted, and two pathological experts identified the relevant pathological type of liver biopsy tissues.

**Figure 2:**
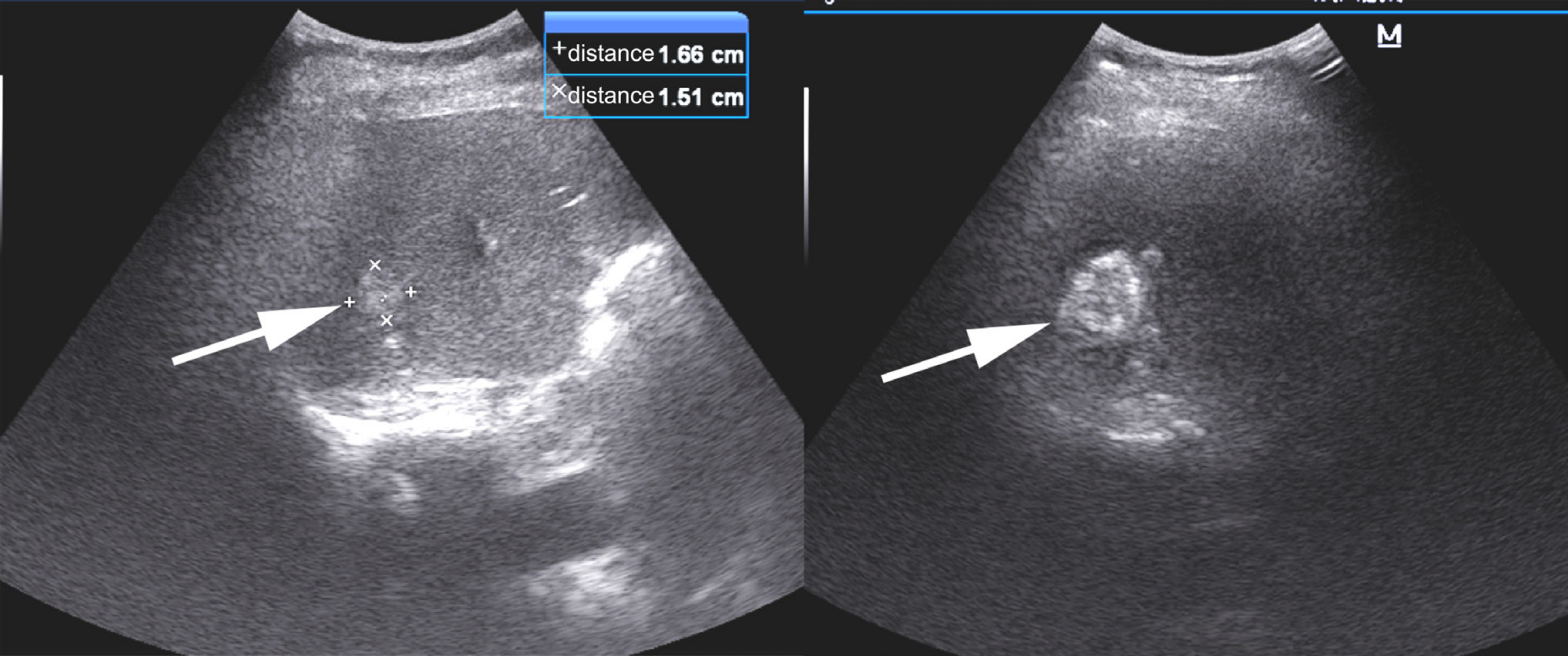
Procedure of MWA using ultrasound imaging The white arrow indicated the potential lesions in the liver. At the same time, we calculated the diameter of tumors as shown in the right and upper part of images.

From Jan 2012 to Jan 2014, 62 CRLM patients underwent clinical treatment of CRLM at our department. Firstly, the liver biopsy was carried out before the treatment, and then the type of pathological samples were defined by two sophiscated pathology doctors (Figure 1). After the diagnosis of CRLM, MWA was conducted in 45.2% (28/62) of CRLM patients as described above and shown in Figure 2. Patient demographics are shown in Table 1. 34 cases of patients underwent hepatectomy. In the patient cohort, 46.8% (29/62) of all patients were female, and the median age was 60 years. Nearly all patients received either preoperative and/or postoperative chemotherapy. In addition, single tumor account for 64.5% of all tumors, and showed no differences from multiple tumors in both groups (*p* = 0.973). The pathological type of most tumors was low/mild differentiated in MWA (85.7%) and resection (82.4%) group. Most importantly, there was no significant difference between treatment and control cohort in tumor size (*p* = 0.487), liver metastasis time point (*p* = 0.368), Child-Pugh (*p* = 0.652) or pathological type (*p* = 0.884). These results suggested that the baselines in both group has no differences.

**Table 1:** Patient demographics and baseline characteristics

Indicators	Type	MWA (*n*= 28)	Resection (*n* = 34)	*Χ*^2^	*P* value
Gender	Male	15	18	0.002	0.961
	Female	13	16		
Age (years)	≤60	16	28	4.736	0.030
	>60	12	6		
Number	1	18	22	0.001	0.973
	2~3	10	12		
Size	≤3 cm	14	20	0.483	0.487
	>3 cm	14	14		
Child-Pugh	A	20	26	0.204	0.652
	B	8	8		
Metastasis time	Heterochronism^a^	18	18	0.812	0.368
	Synchronism^b^	10	16		
Pathological type	Low differentiation	10	13	0.247	0.884
	Mild differentiation	14	15		
	High differentiation	4	6		

a: CRLM occurred at less than 6 months following the CRC resection.

b: CRLM occurred at more than 6 months after the CRC resection

### Major adverse reactions following MWA

**Figure 3:**
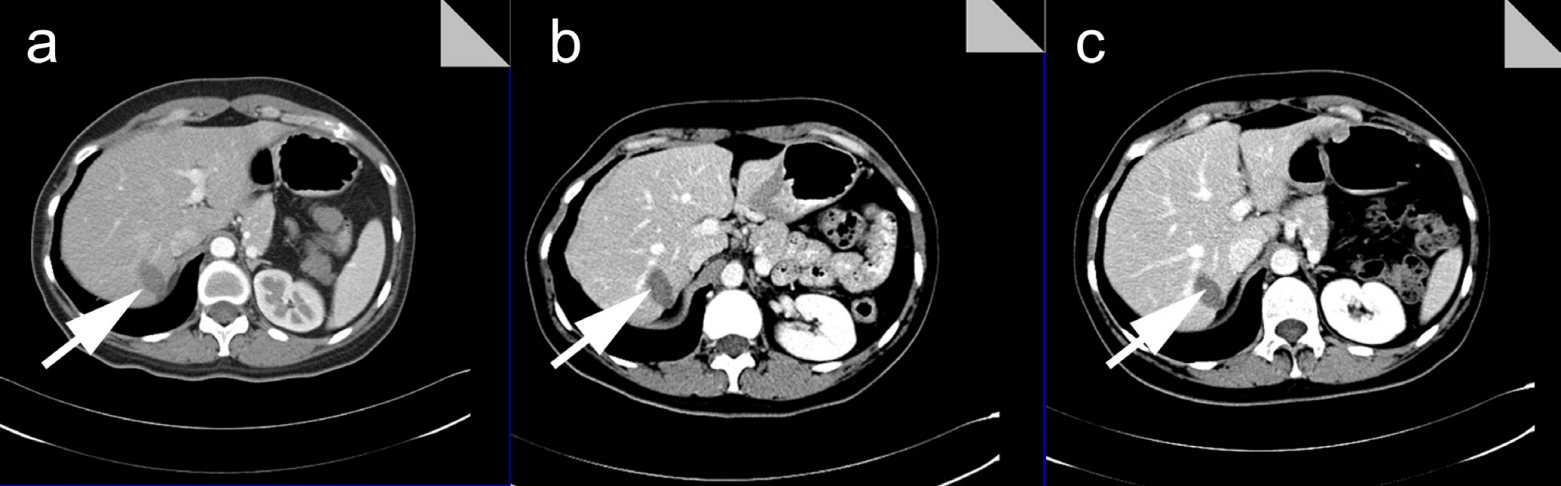
Post-operative CT imaging in a cohort of MWA patients **a**. After one month post-treatment with MWA, the CT imaging showed the low intensity of lesions without peripheral enhancement; **b**. After three months post-treatment with MWA, the CT imaging showed the low intensity of lesions without peripheral enhancement; **c**. After six months post-treatment with MWA, the CT imaging showed much lower intensity of lesions without peripheral enhancement than ever.

After three days of MWA treatment, all patients in this trial were subjected to the contrast-enhanced ultrasound in order to identify the inactivated status of the liver tumor, with which we can determine whether other potential treatments were necessary. Following the identification of ultrasound, alterations in patient pain grade, bogy temperature, detection of routine blood and liver function tests were further identified. And then the patients were examined using enhanced CT/MRI or ultrasound imaging every 3 months following our therapy (Figure 3).

According to detection results, common severe complications were not found in the MWA group, while 9 cases of patients in the resection group was observed with mild or severe complications. Following the treatment of ablation, adverse reactions were mainly recorded as regional pain (18 cases), bradycardia (1 case), fever (1 case) and hepatic insufficiency (13 cases). The pain grade was recorded between the 3th and 6th degree following treatment in 18 patients, among whom three cases accepted the administration of morphine (10mg, i.m.). The bradycardia derived from vagal excitation, and then the atropine (0.5mg, i.v.) was used to alleviate the symptom. Body temperature of one patient was more than 38°C, with total white blood count (11.2×10^9^/L) and neutrophil cell proportion (81%). Thus, relevant symptomatic treatment including anti-infection and clinical observation was conducted to treat patients. Additionally, the increase of AST and ALT level indicated 13 patients suffered from hepatic insufficiency. Relevant hepatic protectants were used to alleviate hepatic functions of patients.

As for liver resection group, we found pleural effusion (4 cases) and perihepatic effusion (2 cases), and then we transfused the albumin and regulated the balance of electrolyte. Besides we observed a patient with periohepatic abscesses, and carried out the drainage for abscesses to alleviate the symptom. During the hospitalization, one patient developed cholangitis, and a patient had wound infection. Thus, the relevant anti-infection therapy was conducted.

Finally, we summarized the hospitalization time and costs, and found that the mean hospitalization duration of patients with MWA is 5.9±0.9 d, which is significantly different from control (11.8±6.9 d) (*p* < 0.001). Furthermore, the average costs of patients with MWA is (29±5) thousand RMB, however, the control needed to spend (55±8) thousand RMB on liver resection, which has obvious differences from MWA group (*p* < 0.000) (Table 2).

**Table 2:** The hospitalization time and costs in both group

group	Time (d)	Cost (thousand)
MWA (*n*=28)	5.9±0.9	29±5
Resection (*n*=34)	11.8±6.9	55±8
T value	-4.487	14.949
P value	0.000	0.000

### Survival status

**Figure 4:**
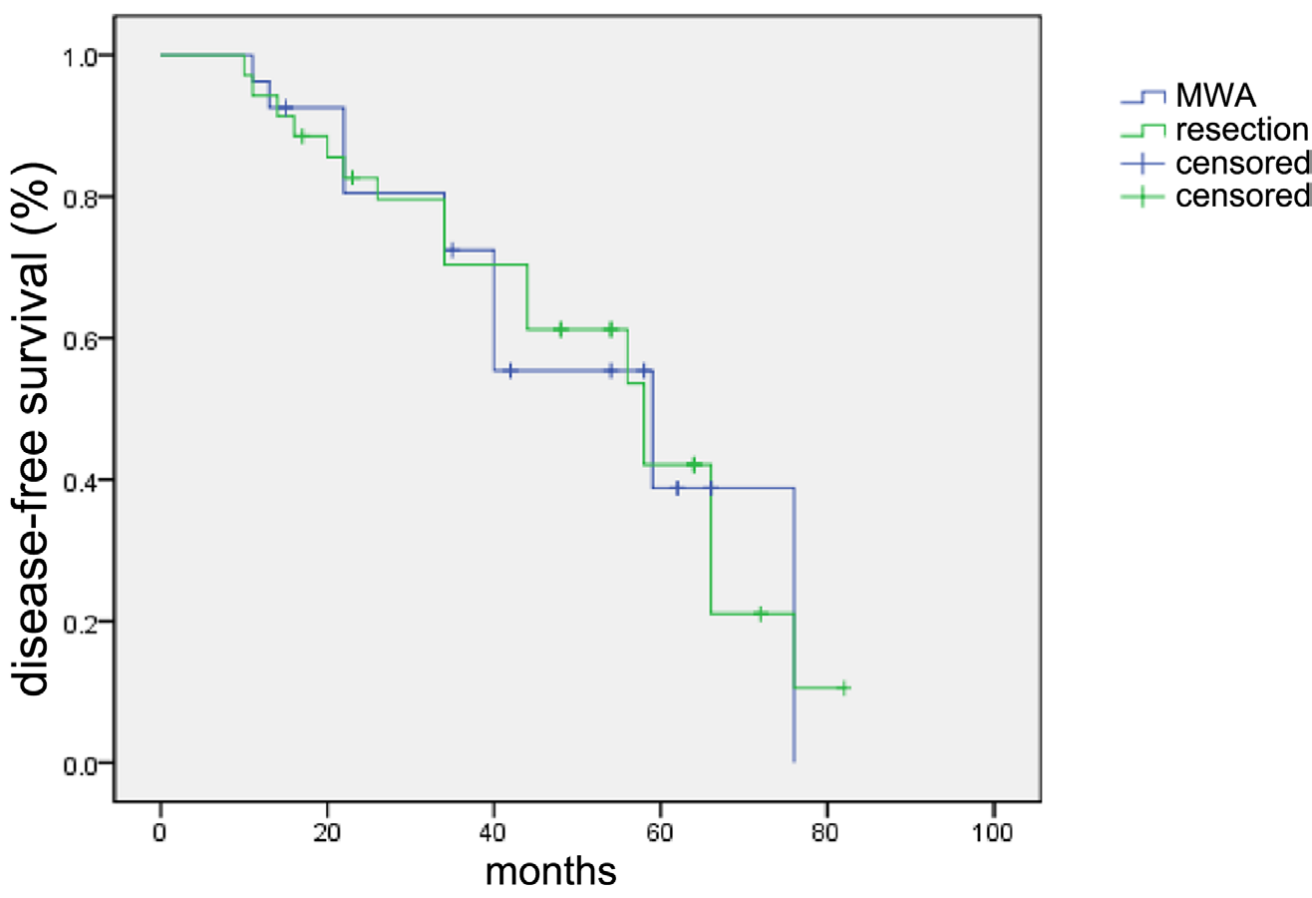
Disease-free survival curve for 28 patients in the MWA treatment group and 34 patients in control group

**Figure 5:**
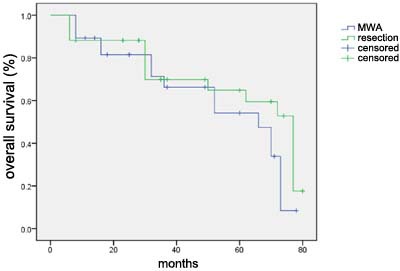
Overall survival curve for 28 patients in the MWA treatment group and 34 patients in control group

In our study, no patient was censored during the observation period. The median follow-up duration was 55 months. Kaplan-Meier analysis was used to estimate the survival status and difference of the two groups (Figure 4, Figure 5). Log-rank test was also used to compare the survival difference of the two groups. Our results indicated that the difference in overall survival and disease-free survival had no statistical significance (χ2 = 0.307, *p* = 0.580; χ2 = 2.010, *p* = 0.156; respectively), suggesting that there was no obvious difference of prognosis in the MWA and control group in the treatment of CRLM patients.

Besides, we summarized potential advantages and disadvantages of MWA therapy as shown in Table 3. Generally, patients with multiple liver metastases that were limited into one specific region or patients with large solitary tumor mass in the liver should undergo liver resection as the first option. Under some conditions, MWA therapy can play an important role in treating patients with small and scattered metastatic tumors in the liver.

**Table 3:** Main potential advantages and disadvantages of MWA

Potential advantages	Potential disadvantages
Good effects on tumors larger than 3 cm Less heat sink effect Use of multiple probes at one time No grounding pads required	Little efficacy data Little safety data Potential injury to critical tissues or organs Variability in MWA devices

## DISCUSSION

According to recent reports, colorectal cancer has a high incidence, and nearly 50% of colorectal cancer patients are likely to develop distant metastases to liver [12]. In recent years, the putative and traditional way to manage patients with CRLM is operative resection or excision., after which the 5-year survival rate can be up to nearly 40%, while only 20% of CRLM patients can be effectively and completely attenuated [13]. Previously patients with resectable CRLM were identified to survive for another 10 months than those without operative resection. With the advent of chemical and novel biological target drugs, the survival periods of CRLM patients has been reported to be increased into about 20 months [14]. As known to all, chemotherapy has some potential adverse effects, and the basic conditions of CRLM patients generally will be serious. Thus minimally invasive treatment will be recommended as a new and reasonable option for a majority of CRLM patients who did not endure liver resection.

The current studies on effects of MWA on CRLM patients demonstrated that MWA is a safe and effective way to treat patients with technically unresectable CRLM, and MWA can establish local disease control in some diseases. According to previous reports, in a cohort of 43 patients, three years overall survival of patients undergoing MWA alone was 32%, and three years overall survival of patients with combined ablation/resection was 45%; Besides, the 3-year disease-free survival rates were 31% and 8% in MWA and resection groups respectively [15]. Other reports also demonstrated that MWA was closely related to the improved survival rate in patients with unresectable CRLM than chemotherapy [16]. In addition, MWA was also reported to offer a larger, more homogenous and uniform stage for treatment of CRLM patients [17, 18]. In this work, cccording to detection results, common severe complications were not found in the MWA group, while 9 cases of patients in the resection group was observed with mild or severe complications. Following the treatment of ablation, adverse reactions were mainly recorded as regional pain (18 cases), bradycardia (1 case), fever (1 case) and hepatic insufficiency (13 cases). The pain grade was recorded between the 3th and 6th degree following treatment in 18 patients, among whom three cases accepted the administration of morphine (10mg, i.m.). The bradycardia derived from vagal excitation, and then the atropine (0.5mg, i.v.) was used to alleviate the symptom. Body temperature of one patient was more than 38°C, with total white blood count (11.2×10^9^/L) and neutrophil cell proportion (81%). Thus, relevant symptomatic treatment including anti-infection and clinical observation was conducted to treat patients. Additionally, the increase of AST and ALT level indicated 13 patients suffered from hepatic insufficiency. Relevant hepatic protectants were used to alleviate hepatic functions of patients. Finally, we summarized the hospitalization time and costs, and found that the mean hospitalization duration of patients with MWA is 5.9±0.9 d, which is significantly different from control. Furthermore, the average costs of patients with MWA is (29±5) thousand RMB, however, the control needed to spend (55±8) thousand RMB on liver resection, which has obvious differences from MWA group. These results indeed demonstrate the safety and effectiveness of MWA.

This study has its limitations, including retrospective and prospective design, and small number of patients. Additionally, follow-up periods of patients were limited, which affected our analysis of long-term survival data. In our study, no patient was censored during the observation period. The median follow-up duration was 55 months. Our results indicated that the difference in overall survival and disease-free survival had no statistical significance, suggesting that there was no obvious difference of prognosis in the MWA and control group in the treatment of CRLM patients. Finally randomized and double-blind trials should be conducted to determine the role of MWA in treatment of CRLM patients.

In conclusion, MWA has been identified to be a safe and effective way to treat CRLM patients. In some conditions, liver resection is also recommended as the first choice to treat CRLM patients. We can assume that MWA combined with liver resection or hepatectomy should be a reasonable and effective way to manage CRLM patients.

## MATERIALS AND METHODS

### Patient inclusion

A retrospective review was conducted on data acquired from institutional review board-approved, retrospectively acquired electronic medical records at Affiliated Hospital of Shandong Academy of Medical Sciences (Jinan, Shandong). Inclusion criteria were conducted as below: (1) tumor diameters of < 5 cm for single liver tumors; (2) tumor diameters of < 3 cm for 2-3 tumors; (3) no invasion of the blood vessels, bile duct and adjacent organs; no distant metastasis; and normal coagulation; (4) KPS score 1-2, and no any contraindications about surgery and microwave; (5) The complete ablation and radical resection were identified based on image data after the patients underwent MWA and surgical resection. According to criteria, we included 62 cases of patients who underwent MWA or hepatectomy of colorectal cancer metastases from Jan 2012 to Jan 2014 at our care center. Three surgeons performed all of the operations over this time period. Preoperative patient characteristics such as age, gender, carcinoembryonic antigen level, history of diabetes mellitus, coronary artery disease, pulmonary disease, and smoking were recorded and analyzed, as well as whether preoperative or postoperative chemotherapy and/or radiation was administered. Postoperative outcomes such as severity of complications (using the Clavien-Dindo classification system), and length of hospitalization were analyzed.

### MWA procedure

All necessary examinations were performed prior to the treatment, and signed informed consent was obtained from each patient. Liver lesions were clearly displayed upon two-dimensional ultrasound, and if the image was not clear, it was located by contrast-enhanced ultrasound. All the patients were treated with intravenous anesthesia, a cooled-shaft microwave system (KY-2000; Kangyou Medical, Nanjing, Jiangsu, China), two coaxial cables and two water-pumping machines, which could simultaneously drive two 15-gauge polytetrafluorethylene-coated cooled-shaft antennae. The generators were able to produce 1-100 W of power at 2,450 MHz. Three types of antennae, with 0.5, 0.7 and 1.1 cm tips, were chosen according to the size of the tumor, diameter < 2, 2-3 and > 3 cm, respectively. A thermal monitoring system with 21-gauge thermocouple needles was equipped in the MWA machine. These needles function by monitoring the real-time temperature percutaneously at a specified location.

### Imaging evaluation

Preoperative assessment of tumors consisted of one or more of the following: computed tomography (CT) scan of the chest/abdomen/pelvis, magnetic resonance imaging (MRI), and/or positron emission tomography (PET) CT. Intraoperative ultrasound was used to identify and confirm lesions, as well as to aid in operative planning. MWAs were performed with the Valley Lab© system.

### Follow-up

Following three days of treatment, all patients were examined by contrast-enhanced ultrasound to determine the inactivated condition of the tumor and to establish whether any supplementary treatment was required. Subsequent to this, changes in the pain grade and temperature, and results of routine blood and liver function tests were observed. The patients were examined by enhanced computed tomography (CT)/magnetic resonance imaging (MRI) or ultrasound imaging every 3 months after treatment. Recurrences were identified *via* postoperative imaging, both systemic and/or local. Local recurrence was defined by a radiologically suspicious lesion at or adjacent to the prior site of ablation. In addition, complications were divided into mild and severe degree. The mild complications involve clinical observation combined with a little treatment intervention, besides the severe complications involve more clinical treatment, potential concurrent diseases, and even death. A follow-up study was performed and lasted up to 60 months.

### Statistical analysis

Groups were compared using the unpaired Student's *t*-test for continuous variables and using Fisher's exact test or the chi-square test for categorical variables. Kaplan-Meier survival estimates were performed to assess both overall and disease-free survival. Disease-free survival was defined as the time to first recurrence. Overall survival was based on the total length of patient follow-up. Univariate analyses were performed using systemic and local recurrence as dependent variables. Values of *p* < 0.05 were considered statistically significant. Statistical analyses were performed using SPSS version 17.0 software.
